# Self-reported poliomyelitis vaccination and documentation in adults indicates high uptake: a digital German epidemic panel, December 2024

**DOI:** 10.1186/s12889-025-24865-9

**Published:** 2025-10-16

**Authors:** Robyn Kettlitz, Manuela Harries, Seba Contreras, Jannik Reinecke, Maren Sophia Wieder, Thomas von Lengerke, Stefanie Castell, Berit Lange, Carolina J. Klett-Tammen, PCR-4-ALL study group, Xavier Casadevall i Solvas, Emine Kayahan, Mariam Mohamed Abdelsatta Bayoumi, Gregor Fritz, Zhiyuan Ma, Jeroen Lammertyn, Dragana Spasic, Lorenz Van Hileghem, Robin De Groote, Javier Martinez-Picado, Elisabet Fernández-Rosas, Sara Morón-López, Maria C. Puertas, Maria C. Garcia-Guerrero, Catia Nicodemo, Alessandro Bucciol, Stefano Landi, Chiara Leardini, Giulia Montresor, Khalidwa Shomali, Isti Rodiah, Felix Jenniches Helmholtz, Daniel Alexander Schulze, Vanessa Melhorn, MuSPAD study group, Monika Strengert, Alex Dulovic, Nicole Schneiderhan-Marra, Jana-Kristin Heise, Gérard Krause, Pilar Hernandez, Daniela Gornyk, Monike Schlüter, Tobias Kerrinnes, Gerhard Bojara, Kerstin Frank, Knut Gubbe, Torsten Tonn, Oliver Kappert, Winfried V. Kern, Thomas Illig, Norman Klopp, Gottfried Roller Michael Ziemons

**Affiliations:** 1 Department of Epidemiology, Helmholtz Centre for Infection Research, Brunswick, Lower Saxony Germany; 2 PhD Programme “Epidemiology”, Brunswick, Hanover Germany; 3 Max Planck Institute for Dynamics and Self-Organization, Göttingen, Germany; 4 Hannover Medical School, Department of Medical Psychology, Hannover, Germany; 5 German Center for Infection Research (DZIF), Partner Site Hannover-Braunschweig, Brunswick, Germany

**Keywords:** Digital epidemiology, Vaccination uptake, Population immunity, Vaccination records, Infectious diseases, Poliomyelitis

## Abstract

**Background:**

On 12 December 2024, the Standing Committee on Vaccination (STIKO) recommended universal polio catch-up vaccination for children and adolescents up to 16, urging parents to check their children’s immunization status following detections of vaccine-derived poliovirus in wastewater. The Robert Koch Institute (RKI) also advised healthcare professionals to ensure vaccination coverage in priority groups. Regional health authorities, called on all citizens to review their vaccination records to address any immunization gaps. We investigated vaccine uptake (documented / recalled) to improve estimates of immunity against poliovirus among the German population and gain insights into the proportion of undocumented vaccines.

**Methods:**

We conducted a survey in December 2024 using the eResearch System PIA (Prospective Monitoring and Management—App) to collect data on self-reported vaccine uptake among a German cohort. We calculated the frequency of vaccinations that were documented and undocumented, as well as the types of vaccines and the number of doses received. Vaccination status was classified as received ≤ 2 doses versus ≥ 3 doses of any polio-containing vaccine. We applied survey weights to calculate frequencies according the general German population (by age, sex, region) and logistic regression to examine the relationships between the vaccinations that were not documented but recalled, and the factors associated with these undocumented vaccinations.

**Results:**

Among 1,124 participants who completed the survey on vaccination uptake, 1,097 (96.9%) participants stated to have a vaccination record. A total of 823/1,124 (74.3%) reported having a vaccination record, where at least one poliomyelitis vaccine was documented, whereas 233 (19.0%) participants recalled at least one poliomyelitis vaccination without documentation or vaccination record. Of 1,124, 68 participants (6.7%) did not report any polio vaccination neither documented nor recalled without documentation. Among the 823 participants with documented vaccination and at least one vaccination, 592 (75.1%) received at least three doses of a poliomyelitis vaccine, with a decline in older age groups, less than three doses were reported by 164 (17.6%), and the remaining 7.3% (*n* = 67) did not have information on the number of doses administered. Of 2,768 documented vaccine doses, 898 (29.9%) were oral poliovirus vaccines (OPV) and 704 (26.2%) were inactivated poliovirus vaccines (IPV). In 1,166 vaccines (43.9%), the type could not be derived by the participants from the vaccination record. The odds of having a recalled vaccination (not documented) was higher in male and the older age groups compared to females and younger participants.

**Discussion:**

We found similar poliomyelitis vaccination uptake compared to other data sources e.g., of the Robert Koch Institute (RKI). Vaccine-derived immunity to poliomyelitis may be underestimated based on vaccination records only. There is a need to address potential gaps in health literacy and vaccination documentation. Efforts should be made to conduct continuous seroprevalence surveys in the population in response to emerging public health threats and deduce parameters to inform modelling infection dynamics in specific outbreak scenarios.

**Trial registration:**

The PCR-4-ALL cohort was registered in the German Clinical Trials Register on the 3rd of September 2024 (DRKS00034763).

**Supplementary Information:**

The online version contains supplementary material available at 10.1186/s12889-025-24865-9.

## Background

By the end of 2024, wastewater surveillance detected evidence of circulating vaccine-derived poliovirus (cVDPV2) in several countries where wild type polioviruses had been eradicated, including Germany and other countries in the EU [1–3]. In 2020, 1,418 cVDPV2 cases were reported in four of the six WHO geographical regions [4]. The source of cVDPV2, the live attenuated oral poliovirus vaccines (OPV), had a crucial role in reducing global poliomyelitis cases by over 99% since their introduction [4, 5]. In Germany, the German Democratic Republic (GDR) implemented widespread polio vaccination campaigns starting in 1960, including mandatory vaccinations at home and in schools. These campaigns quickly achieved high coverage rates [6–8]. In contrast, the Federal Republic of Germany (FRD) began oral polio vaccination in 1962, mainly through voluntary vaccinations and public vaccination campaigns, which resulted in slower vaccination uptake [7–10]. Advantages of OPV over other vaccine types include lower costs, its ability to enhance intestinal immunity, and its ease of administration in the form of oral drops and storage [11, 12]. Yet, OPV is no longer used in the EU because of its risk of vaccine-associated paralytic poliomyelitis (VAPP) [4, 5], especially if the levels of immunity against poliovirus in the population are low [13]. Consistently, OPV continues to be an effective and rapidly deployable vaccine, whereas in countries with low incidence, the relative risk of VAPP caused by OPV is greater than the lower infection risk [4, 14]. In Germany the individual vaccination uptake is typically documented on paper-based records, according to § 22 [1] of the Infection Protection Act (IfSG) [15].

Since 1998, only inactivated poliovirus vaccine (IPV) is recommended for immunization in Germany [9]. Until 2020, in Germany a 3 + 1 vaccination schedule for primary immunization was recommended. In June 2020, the German Standing Committee on Vaccination (STIKO) introduced the simplified 2 + 1 schedule to maintain comparable protection while reducing the number of doses [16]. IPV is an effective vaccine, approximately 90% of vaccinated are immune after two doses and at least 99% are immune against poliovirus after three doses [17]. However, this vaccine does not provide high levels of immunity in the intestine; individuals vaccinated with IPV can still be infected (in the gut) and shed the virus into the environment [14, 18]. As long as poliovirus is not eradicated globally, it can be re-imported, so vaccination efforts have to be in place also in countries with a low incidence of poliovirus infections [19]. To estimate the levels of immunity among the population, we need further information about early life vaccination including vaccine types, which is not captured in most available data. On 12 December 2024, the Standing Committee on Vaccination (STIKO) recommended universal polio catch-up immunization for children and adolescents up to 16 years and urged parents to verify their children’s vaccination status with their physician in response to the vaccine-derived poliovirus detections in wastewater [16]. The Robert Koch Institute (RKI) also called on healthcare and public health professionals to review and complete immunizations for priority groups [2]. In addition, local health authorities and ministries across Germany, e.g. the Ministry of Labor, Health and Social Affairs of North Rhine-Westphalia (MAGS NRW) and the Lower Saxony Public Health Agency (NLGA), encouraged all citizens to review their vaccination records and consult medical professionals to close any immunization gaps [20, 21].

In this study, we aim to identify poliomyelitis vaccination gaps in the German population. To this end, a digital cohort [“Impact and viability of a novel mass PCR testing method as a pandemic-fighting strategy” (PCR-4-ALL) cohort; see Methods] was surveyed to ascertain poliomyelitis vaccination recall or documentation in vaccination records, including vaccine type when available on 19th of December 2024. Thereby, we sought to quantify the fraction of the population that seem not to have had at least ≥ 3 doses of any polio-containing vaccine. As it is currently not possible to retrieve vaccination status from common seroprevalence studies and there are no digital vaccination records available to estimate the vaccine-induced immunity in the German population (including undocumented cases).

## Methods

### Study population & design

**Fig. 1:**
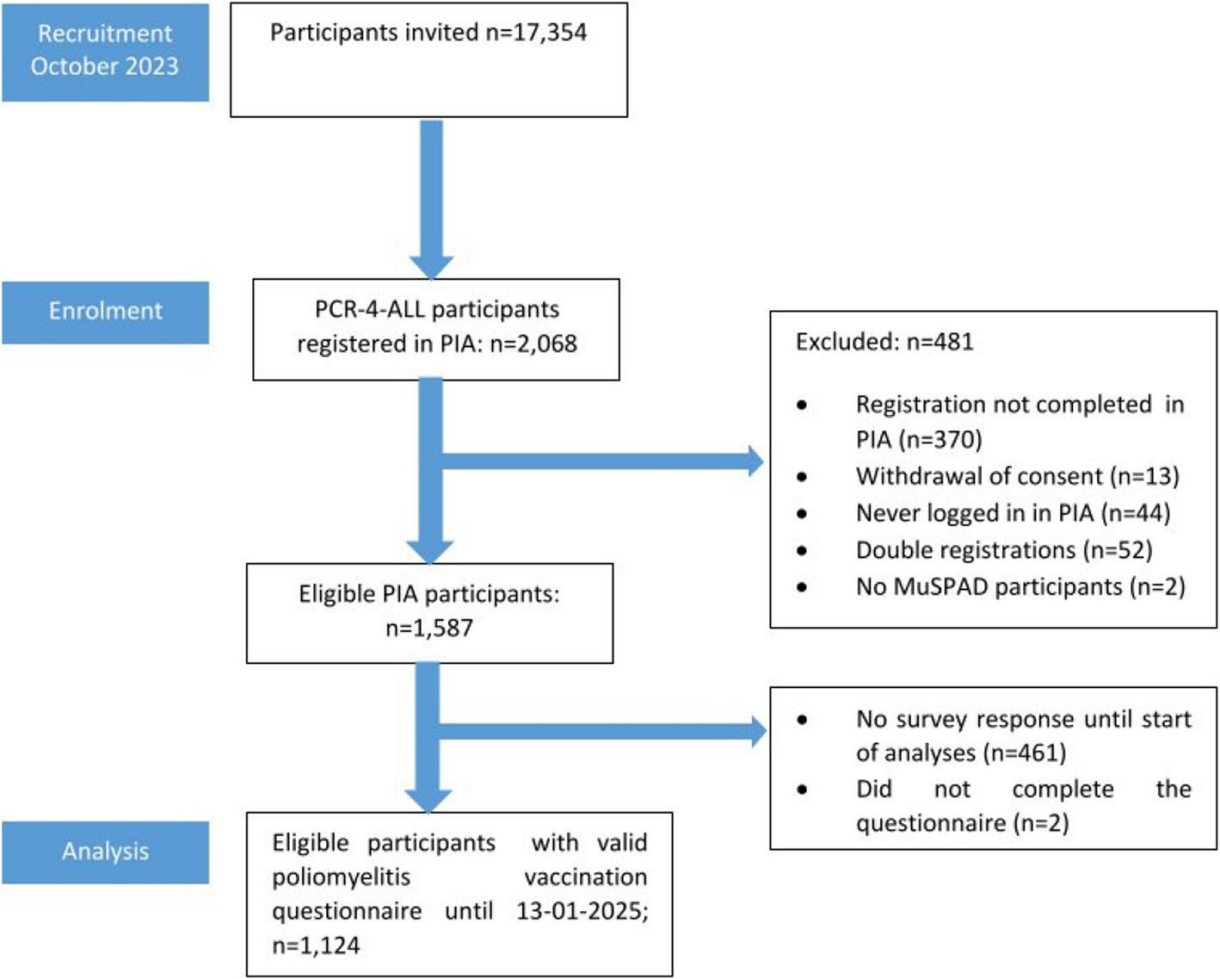
Flow chart of recruitment and study sub-cohort PCR-4-ALL

We created and applied a questionnaire to assess poliomyelitis vaccination status among an standing epidemic panel (PCR-4-ALL cohort) [22]. The study population is a sub-cohort of the “Multilocal and Serial Prevalence Study of Antibodies against SARS-2 Coronavirus in Germany” (MuSPAD), a population-based (sero)epidemiological study including 25,712 randomly recruited (by age and gender) participants from eight regions/districts (Kreise) of Germany (Reutlingen, Freiburg, Aachen, Magdeburg, Osnabrück, Chemnitz, Greifswald, Hannover) in 2020 (more detailed regional distribution, see Supplement Table 1), originally initiated to determine the prevalence and incidence of antibodies against respiratory infections [23] and, then transformed to an epidemic panel to answer emerging questions on infectious diseases. The inclusion criteria was to participate in MuSPAD and having registered a valid e-mail address (*n* = 17,345). The sub-cohort was finally constituted by the subset of those that registered in the eResearch system *Prospective Monitoring and Management—App* (PIA) [24, 25] by October 2023 (*n* = 2,063). The sub-cohort started on October 2023, with questionnaires on respiratory symptoms, vaccination behavior, and self-applied rapid tests for different types of infection (Fig. 1).

### Assessment of self-reported poliomyelitis vaccination

We designed and programmed in PIA a self-administered questionnaire on poliomyelitis vaccination status, distributed to participants of PCR-4-ALL on December 19th, 2024. This happened a week after the Standing Committee on Vaccination (STIKO) called on parents and doctors to review the vaccination records of children and potentially vulnerable people who were at risk of being under-vaccinated. Prior to its release, the questionnaire underwent a cognitive pre-test for validation and adapted accordingly. It was then made accessible through the PIA system, which could be reached either through a dedicated smartphone app or web application via web browser. To encourage higher participation rates, additional reminders were sent out in the form of push notifications and follow-up emails. Registered participants were asked to indicate whether they possessed a vaccination record and to self-report their poliomyelitis vaccination status based on documentation provided or, if unavailable, from memory (recalled vaccination). We also asked about the vaccine type (OPV versus IPV) and the number of doses received including the date. Responses were considered until January 13th, 2025. The codebook of the questionnaire can be retrieved from elsewhere (doi.org/10.5281/zenodo.15121476).

### Outcome and variable definitions

Since 2020, immunization recommendations in Germany related to poliomyelitis vaccination is the 2 + 1 schedule for term infants, which consists of three combination vaccine doses administered within the first year with IPV [26]. For preterm infants, the 3 + 1 schedule is recommended, with completion defined as four or more doses of IPV [26]. Between 1998 and 2020, the 3 + 1 schedule was also the standard recommendation for all infants in Germany [10]. In the time when Germany was divided into the FRG and the GDR, various OPV formulations, including monovalent and trivalent OPV, were used. In these cases, higher total dose numbers were typically recommended [27]. Individuals born between 1960 and 1998 were considered “fully immunized” after receiving at least four doses of trivalent OPV, while individuals born in the GDR were expected to receive five doses (three monovalent OPV and two trivalent OPV) [27]. However, some limitations in our data prevented us from applying these national definitions with precision. These limitations included missing or illegible vaccination entries in the mostly handwritten vaccination records, inability to distinguish between monovalent and trivalent OPV, and the absence of reliable information on participants’ birth region or migration history. Therefore, we adapted the WHO/UNICEF Pol3 indicator, which defines a receipt of at least three doses of a polio-containing vaccine in 1-year olds, and applied it for the whole life course [28], allowing us for internal consistency and international comparability of immunization coverage. Three doses of IPV provide at least 99% protection against paralytic poliomyelitis [17], while a three-dose series of trivalent OPV results in protective seroconversion in at least 95% of recipients [29, 30]. Participants with a record that stated a poliomyelitis vaccination, but gave no information about the numbers of vaccination were categorized as no report on number of doses. Regarding the types of vaccine, we asked about OPV (with different wordings common in vaccination records, i.e. "oral polio vaccine (OPV) Sabin strain, types 1, 2, 3"), and IPV (Repevax, Revaxis, Boostrix Polio, Tetravac, Infanrix, Pentavac, IPV Merieux, others), or if the documentation was unclear, poliomyelitis vaccination could not be identified on the vaccination records by the participants (vaccine type unidentifiable). More detailed information on the vaccination were provided in the questionnaire including links to the RKI (Germany’s public health institute). When recording vaccination uptake, we distinguished between documented vaccination uptake, based on vaccination records, recalled vaccination uptake, based on memories not backed-up with vaccination records (recalled), and no vaccination uptake reported, wherever no vaccination was found by participants in their vaccination record and none could be recalled.

### Statistical analysis

Multivariable procedures (logistic regression) were applied to identify odds of recalled vaccinations without documentation versus documented vaccinations. Independent variables were selected due to subject matter knowledge (age, education, region, and gender). For the regression, we analyzed data from all participants who responded (complete case analysis) (*n* = 1,043) and excluded those with missing values in the variable education (*n* = 13). For the regression model, we stratified the education level by International Standard Classification of Education (ISCED). Level 3 equals German “Abitur” vs. lower, because of the low number of observations within those with an education certificate after 9 years (*n* = 21). Statistical significance was set at p < 0.05 and estimates (odds ratios) included the 95% confidence intervals (CI). Data was analyzed with the statistics software R (Version 4.13) [31]. The sample was weighted to match the German adult population distribution by gender (female/male), age groups (< 40; 40–56; ≥ 60 years) and region (West/East Germany), based on 2023 DESTATIS data [32–34]. Each individual *i* was assigned a weight (W_i_ = P_i_ Germany/P_i_ study sample), where P_i_ Germany is the proportion of the German adult population in person i’s age–sex–region stratum and P*i* study sample is the corresponding unweighted sample proportion. We then estimated weighted proportions and 95% confidence intervals with the survey package in R [35]. We used OpenAI's ChatGPT v2 to review and refine the R code for improved structure and functionality [36]. For clarity and precision, the English language parts of this manuscript have been edited using DeepL Pro. The authors checked the output for correctness and took responsibility for it [37].

### Data protection, ethics, consent

The study complies with the General Data Protection Regulation and the Federal Data Protection Act and Recommendations for Ensuring Good Epidemiological Practice of the German Society for Epidemiology e.V. [38]. Ethical approval for MuSPAD was obtained on June 21, 2020 by the Ethics Committee of the Hannover Medical School (No 9086_BO_S_2020) including the amendment for PCR-4-ALL in 2023. The PCR-4-ALL cohort was registered at the German Clinical Trials Register on the 3rd of September 2024 (DRKS00034763). During self-registration in PIA, participants gave informed consent in the app, and could also withdraw it there. The study was performed in accordance with the Declaration of Helsinki. This study was conducted in accordance with the STROBE guidelines (Strengthening the Reporting of Observational Studies in Epidemiology) [39].

## Results

### Characteristics of study population

In October 2023, we invited 17,354 participants in the MuSAPD study, of whom 1,587 were eligible and registered in the PIA eResearch system. A total of 1,124/1,587 (71.8%) participants answered the poliomyelitis vaccination questionnaire. The participants were predominantly women (50.7% [95% CI: 46.9%, 54.5%]), the median age was 57 years [IQR: 46–66, *n* = 1,124]), and the most common age group was 40–59 years (33.1% [95% CI: 29.8%, 36.3%]). According to ISCED, the majority of participants had a higher level of education (level 3 or higher) (74.0% [95% CI: 70.8%, 77.2%]). Nearly all participants (96.9% [95.5%, 98.2%]) reported that they had a vaccination record (Table 1).

**Table 1:** Characteristics of study population

Characteristics	Overall		Recalled and documented vaccination uptake^c^		Documented Vaccination uptake		Recalled vaccination uptake	
	**Total N**^**a**^	**% [95% CI]**^**b**^ ^**−**weighted−^	**Total N**^**a**^	**% [95% CI]**^**2**^ ^**−**weighted−^	**Total N**^**a**^	**% [95% CI]**^**b**^ ^−weighted−^	**Total N**^**a**^	**% [95% CI]**^**b**^ ^**−**weighted−^
Total	1,124	100%	1,056	93.3% [91.4%, 95.2%]	823	74.3% [71.1%, 77.5%]	233	19.0% [16.2%, 21.7%]
Region of Germany^c^								
West Germany	624	84.4% [82.7%, 86.2%]	584	84.3% [82.5%, 86.1%]	455	84.2% [82.2%, 86.3%]	129	84.6% [81.1%, 88.2%]
East Germany	500	15.6% [13.9%, 17.3%]	472	15.7% [13.9%, 17.5%]	368	15.8% [13.7%, 17.9%]	104	15.4% [11.8%, 18.9%]
Age category (years)								
< 40	161	30.2% [26.1%, 34.3%]	148	29.9% [25.7%, 34.2%]	138	35.2% [30.4%, 40.1%]	10	9.1% [2.5%, 15.7%]
40–59	493	33.1% [29.8%, 36.3%]	468	33.4% [30.1%, 36.8%]	375	32.8% [29.1%, 36.6%]	93	35.9% [28.5%, 43.2%]
≥ 60	470	36.7% [33.3%, 40.2%]	440	36.6% [33.1%, 40.2%]	310	32.0% [28.1%, 35.8%]	130	55.0% [47.0%, 63.1%]
Gender								
Male	428	49.3% [45.5%, 53.1%]	398	49.2% [45.3%, 53.2%]	294	46.2% [41.7%, 50.8%]	104	60.9% [53.6%, 68.3%]
Female	696	50.7% [46.9%, 54.5%]	658	50.8% [46.8%, 54.7%]	529	53.8% [49.2%, 58.3%]	129	39.1% [31.7%, 46.4%]
Education								
Lower than ISCED level 3^d^	341	26.0% [22.8%, 29.3%]	309	24.2% [21.0%, 27.4%]	236	22.7% [19.2%, 26.2%]	73	30.1% [22.6%, 37.6%]
ISCED level 3^d^	770	74.0% [70.8%, 77.2%]	734	75.8% [72.6%, 79.0%]	577	77.3% [73.8%, 80.8%]	157	69.9% [62.4%, 77.4%[
Missing/Other	13	-	13	-	10	-	3	-
Vaccination records available								
No	27	3.1% [1.8%, 4.5%]	20	2.4% [1.2%, 3.5%]	NA	NA	20	11.6% [6.3%, 17.0%]
Yes	1,097	96.9% [95.5%, 98.2%]	1,036	97.6% [96.5%, 98.8%]	823	100%	213	88.4% [83.0%, 93.8%]

^a^Total amount of study participants who filled out the questionnaire about poliomyelitis vaccination and were able to match to their MuSPAD

^b^showing the -weighted- (by age, gender, region) frequency and 95% CI

^c^The regions of Germany correspond to the former division into the German Democratic Republic (GDR) and the Federal Republic of Germany (FRG)

^d^classified according to ISCED (International Standard Classification of Education); level 3 equals German “Abitur”

### Estimating vaccination coverage considering documented and recalled vaccination

Overall, we found a self-reported vaccination uptake against poliovirus in 93.3% [95% CI: 91.4%, 95.2%]; *n* = 1,056] in the study population, of which 74.3% (95% CI: [71.1%, 77.5%]; *n* = 823) had at least one documented dose and 19.0% (95% CI: [16.2%, 21.7%]; *n* = 233) recalled having been vaccinated (without vaccination records or undocumented). Analyzing all self-reported vaccinations (documented and recalled) participants, the highest vaccination uptake was documented in the youngest age groups and in women. The highest percentage of recalled vaccinations was found in the older age groups and in men. The highest proportions of missing/unknown data were found in the age groups ≥ 60 years (Supplement Table 3).

### Self-reported vaccination uptake based on frequency and time point of vaccination

**Fig. 2:**
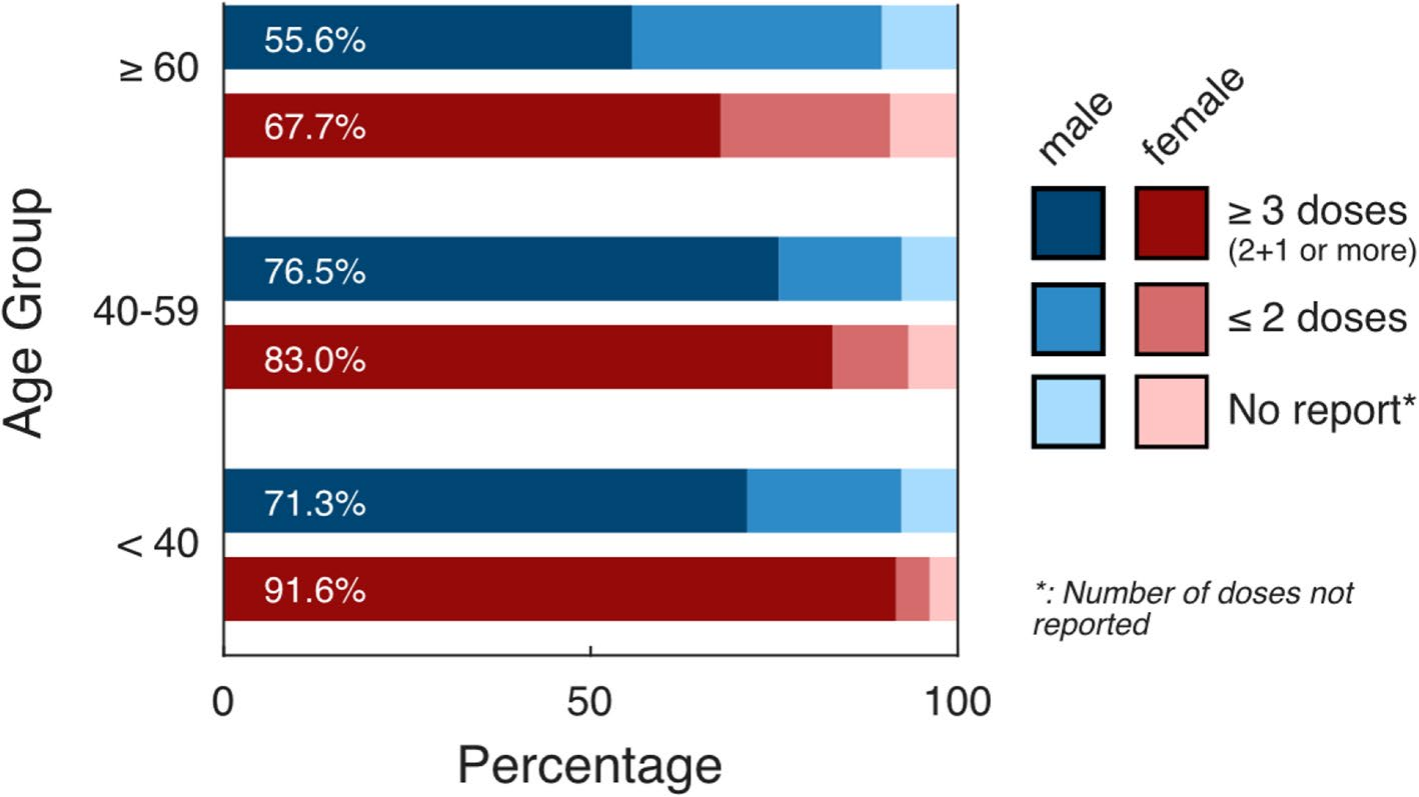
Weighted frequency: Documented poliomyelitis immunization status in the PCR-4-ALL/MuSPAD cohorts based on vaccination record according to the amount of doses (≤ 2 doses/≥ 3 doses); (*n* = 823)

Among the 823 participants with documented poliomyelitis vaccination following the Pol3 indicator, 75.1% (95% CI: [71.3%, 79.0%], *n* = 592) received ≥ 3 doses, 17.6% (95% CI: [14.2%, 20.9%], *n* = 164) received ≤ 2 doses, and the remaining 7.3% (95% CI: [5.0%, 9.6%], n = 67) did not have information on the number of doses administered. When stratifying these trends by age and gender, there is a steady decline in ≥ 3 doses with increasing age and in males compared to female (Fig. 2, Supplement Table 4). For comparison to the 3 + 1 schedule, please the Supplement Fig. 2.

### Type of poliomyelitis vaccine

**Fig. 3:**
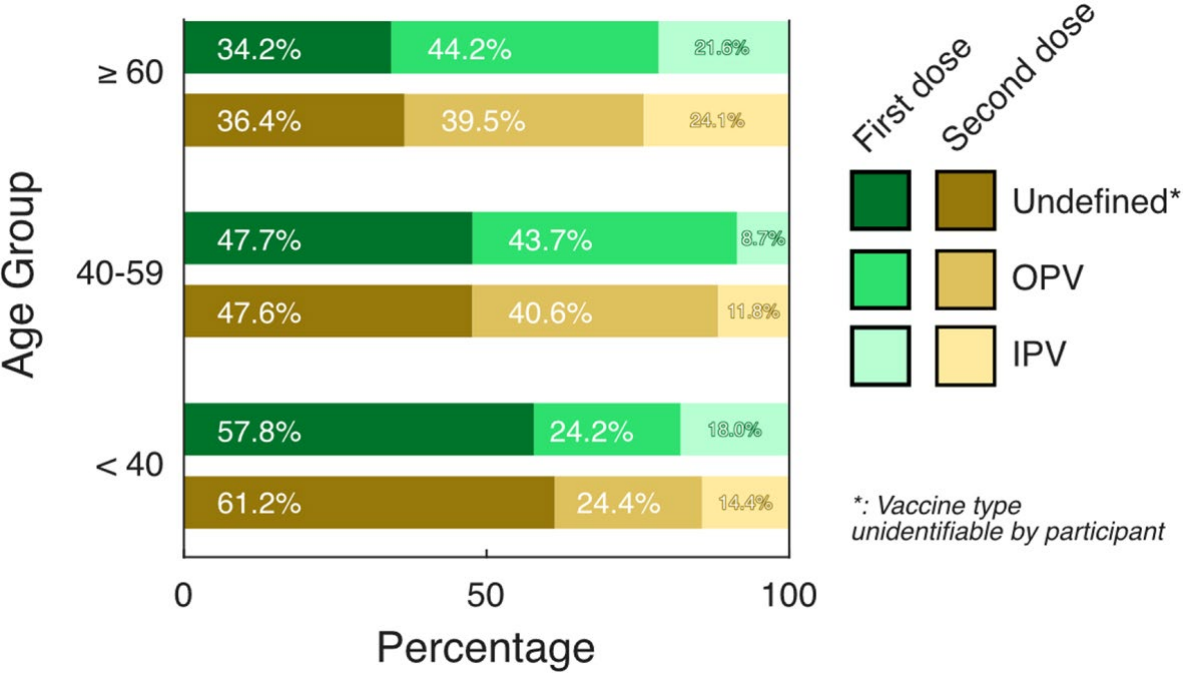
Weighted frequency: Type of poliomyelitis vaccination documented in the vaccination records in the PCR-4-ALL/MuSPAD cohort stratified by age and first and second dose (*n* = 823)

Among the 823 participants with documented poliomyelitis vaccination, 51.1% [95% CI: 46.5%, 55.8%; *n* = 376] reported having received more than five doses, about 17.4% ([95% CI: 13.6%, 21.2%]; *n* = 108) four doses, 12.5% ([95% CI: 9.6%, 15.4%]; *n* = 108) three doses, and two doses 11.7% ([8.7%, 14.6%]; *n* = 107) and 7.3% ([95% CI: 4.8%, 9.7%]; *n* = 57) reported having received one dose. In total, 2,768 vaccine doses were reported to be documented, and OPV was the most frequent vaccine type (29.9% [95% CI: 27.8%, 32.0%]; *n* = 898), followed by IPV (26.2% [95% CI: 24.0%, 28.3%]; *n* = 704), while 43.9% ([41.5%, 46.4%]; *n* = 1,166) stated that the vaccine type was unidentifiable. While OPV is the most documented vaccine type, the proportion of IPV increases within the second dose. Figure 3 shows the distribution of vaccine type given by first and second dose, stratified by age groups and gender. The detailed data can be retrieved from Supplement Table 6–8.

### Determinants for not documented recalled immunization

**Fig. 4:**
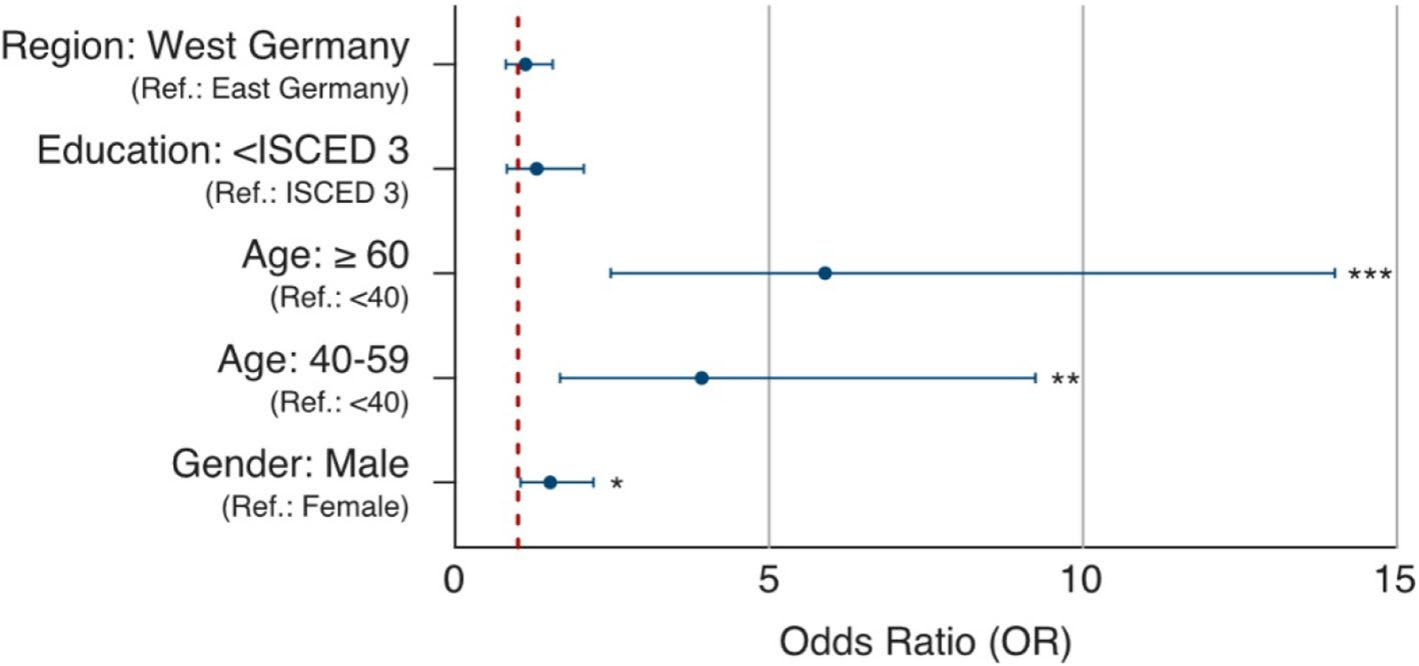
Odds ratio of the associations with an only recalled vaccination uptake (not documented) compared to documented vaccination uptake (*n* = 1,043); education: ISCED = classified according to ISCED (International Standard Classification of Education) level 3 equals German “Abitur”; * *p* < 0.05; ** *p* < 0.01; ***p < 0.001

We studied associations between recalled (not documented) and documented vaccination uptake, adjusting for age, gender, education, and region (*n* = 1,043) (Fig. 4). Higher OR (odds ratios) were found for recalled vaccination against poliovirus in the older age groups compared with the reference group of those under 40 years of age [40–59: OR: 3.93 (95% CI: 1.67–9.24); 60 and above: OR: 5.89 (95% CI: 2.48–14.01)]. The odds of having a recalled vaccination uptake were higher in male [OR: 1.51 (95% CI: 1.04–2.20)], to a lower extent in western Germany compared to eastern Germany [OR: 1.12 (95% CI: 0.81–1.55)] and, in terms of education, with ISCED level lower than level 3 to those participants with ISCED level 3 [OR: 1.30 (95% CI: 0.82–2.05)]. A model summary can be found in Supplement Table 9.

## Discussion

Given the recent and persistent findings of cVDPV2 in wastewaters in several countries where wild type polioviruses have been eradicated, e.g., Germany, quantifying and classifying immunity in the population is timely need. This study provides insights into vaccination uptake against poliovirus infection, with a focus on the role of documentation in vaccination records and the potential for extrapolating cases where documentation is missing or unidentifiable for the general population in the vaccination records. We found that 74.3% of the participants have records of receiving at least one poliomyelitis vaccine (*n* = 823). If we add the people who recalled at least one vaccination and do not have the vaccine documented in the records, the overall vaccine uptake increased to 93.3%, suggesting that at least partial vaccination uptake (in terms of a lack of doses) in this adult study population is rather high. The highest documented uptake was found in the youngest age groups, with a steady decline in the older age groups. This might be because, as people get older, the way vaccines are documented may change, or the probability that the vaccination records had been lost increases. In this sense, age and being male was significantly associated with only having recalled vaccination uptake. Consistently, the oldest age groups and males showed the lowest proportion of receiving ≥ 3 doses. The odds of recalled vaccination uptake did not significantly vary between educational groups, i.e. ISCED levels, which is consistent with the observation that the tendency to carefully weigh up risks and benefits of vaccinations did not differ between these groups [40].

In our cohort, OPV was administered 898 times (29.9%), IPV about 704 times (26.2%), and vaccine type unidentifiable was reported by 1,166 times (43.9%). The proportion of IPV increased during the second dose. Current numbers on vaccination uptake for the birth cohorts 2018 to 2021 in Germany, based on billing data of contracted physicians from the Association of Statutory Health Insurance Physicians, showed that the documented vaccination uptake for poliomyelitis is 96% for dose one and 77% for the fully vaccinated (four doses of vaccine or three doses of vaccine with best-possible intervals), similar to our results in adults [41]. We see in our data, similar to the RKI figures, that vaccination series are often incomplete (i.e., based on documentation) [41, 42]. In Germany, a booster is recommended between ages 9 and 16 after childhood immunization. Routine boosters after 18 are not advised. If primary immunization is given in adulthood, a single booster is recommended 10 years after primary immunization. Additional boosters may be needed for individual at increased risk, e.g., due to travel or occupational exposure [43]. This underlines the need for monitoring vaccine uptake in the older populations for completeness of basic immunization. In addition, our analysis provides individual-level insights into vaccine type combinations and undocumented, recalled vaccinations.

The strength of our rapid survey on poliomyelitis vaccine uptake is the structural readiness of our digital population-based epidemic panel. We have shown the feasibility of quickly assessing the public's self-reported immunity in case of an emerging public health threat. The results indicated that the number of people who got the poliomyelitis vaccine based on vaccine records alone might be underestimating the real uptake level. To further evaluate this hypothesis, the specific antibody levels in those participants would have to be analyzed. Moreover, 43.9% of the doses could not be identified by participants, likely due to incomplete records or limited health literacy. In other situations, such as the recent increase in measles cases in Europe, official guidelines recommend that individuals check their own vaccination status [44]. However, many people may fail to determine their status accurately from their vaccination records, especially when information is missing, incomplete or unclearly documented. To reduce potential misclassification bias and enhance the health-related self-efficacy and health literacy, future studies should compare self-reported vaccination status with evaluations by medical staff. There is a need to help to identify both the reasons why participants are unable to determine their vaccine type and the factors contributing to unclassifiable records.

Our study has some limitations. For example, since only about 12% (2,068/17,354) of the original cohort participated in the newly established digital study, we must consider selection bias. Participants registered in PIA might be likely more health-conscious and interested in research. Furthermore, the poliomyelitis vaccination uptake was assessed by self-reports, with specific characteristics. Besides, we did not consider the time between doses, which may be important in determining whether three or four doses are sufficient for complete uptake, and recall bias needs to be taken into account by the recalled vaccinations. Since the median age in the study population is higher as well as higher educated, i.e., ISCED level 3, than in the general population. This might introduce a bias in that vaccinations are viewed more favorable in this group compared to those with lower education [45]. Subsequently, survey weights were applied based on age, sex, and regional distribution to account for underrepresented groups and improve representativeness of the study sample. However, unmeasured factors e.g. socioeconomic status and migration history, which may still lead to deviations in our study sample from the actual German population, were not taken into account.

Notably, our study did not reveal significant differences in recalled vaccinations between West and East Germany. However, historical events, such as the 1960 mass vaccination campaign in Halle (East Germany), where over 63,000 children were vaccinated against poliomyelitis, may have resulted in incomplete documentation in vaccination records. Furthermore, the East–West Germany comparison in our study is based on participants' current place of residence, rather than their birthplace. This might not accurately reflect their geographical location at the time of vaccination, potentially introducing bias into our results [6]. Still, we chose this approach to give insight into current geographical priorities for, e.g., vaccination campaigns. Previous research has identified potential clustering of lower vaccination uptake in specific contexts, such as certain school types or minority communities [46]. However, our study's limited geographic resolution and lack of subgroup representation precluded an examination of this phenomenon. Investigating these potential clusters could be a valuable avenue for future research, offering insights into targeted interventions to improve vaccination coverage. Another limitation of the dataset is that it does not enable us to determine how participants received IPV vaccinations prior to their inclusion in the routine vaccination schedule in Germany. The absence of information regarding alternative sources of IPV vaccination, such as living abroad or private healthcare, may affect the results and should be considered when interpreting the data. Furthermore, the lack of thoroughly evaluation of historical vaccination campaigns and uptake shows to be problematic also for estimating current immunization status.

Lessons learned highlight the need for a clearer, more user-friendly recruitment and registration process to improve participation in digital cohorts. Investigating reasons for non-participation can help reduce selection bias. To improve data validity and gain deeper insights, future studies should integrate, compare and supplement self-reported vaccination status, documented vaccination records, billing data from health insurance companies or doctors, and serological assessments, possibly using self-sampling. Assessing participant’s health literacy and comparing it with expert evaluations may reveal key gaps in understanding and give the opportunity to enhance existing initiatives. Identifying specific barriers, such as unclear documentation or terminology, can help reduce misclassification in self-reported data. Still, digital surveys in standing epidemic panels give the opportunity, to rapidly assess emergent research questions. Regarding recalling specific vaccinations, polio is a special case, as the accompanying campaign in the beginning of the public health strategy was so widely spread and known and the type of the OPV so special that many people can actually remember, which is not transferrable to other vaccines.

The need for enhancing vaccine uptake over the life-course and especially in the adult and the elderly population has not been addressed successfully in the last years. Our results are consistent with those of other European countries, highlighting the importance of monitoring vaccination uptake, especially with increasing age. It is crucial that future research analyzes how the general population or healthcare professionals responded to the call for (self-)assessment of vaccination status, particularly with the help of information material specifically designed for this purpose [27]. In particular to determine whether health literacy was sufficient to follow the instructions. When developing public health measures against cVDPV2 in communities, risk groups should be given special consideration. Based on the latest results of the wastewater monitoring, it is not yet clear whether these are multiple imports or whether cVDPV2 has already been transmitted locally [47]. To date, no suspected cases of polio have been reported in Germany. Nevertheless, it is necessary that further research prioritizes the validation of the underestimation of vaccine uptake. Our study can contribute to a valid estimation of vaccine-induced immunity and gaps of vaccine uptake (documentation) in specific populations as a basis for modelling infection dynamics in specific outbreak scenarios.

## Supplementary Information

Supplementary Material 1.

## Data Availability

Aggregated data is presented in the figures and tables of the manuscript. Due to data protection concerns, access to the individual-level data is restricted. Upon request anonymized data can be provided. Please contact the authors if you have any questions or require further information.
