# Supplementary material for: Self-reported poliomyelitis vaccination and documentation in adults indicates high uptake: a digital German epidemic panel, December 2024
Source: BMC Public Health. 2025 Oct 16;25:3514. doi: 10.1186/s12889-025-24865-9 (PMC12532904; doi:10.1186/s12889-025-24865-9)
Supplement: Supplementary file 1 — Supplementary Material 1. [file 12889_2025_24865_MOESM1_ESM.docx]

# Supplement

**Supplement Table 1:** Unweighted frequency of the PCR-4-ALL/MuSPAD subcohort

| **Characteristics** | **Counts (n)^1^** | **Frequency [95% CI)**  -unweighted-^2^ |
| --- | --- | --- |
| **Region of Germany** | | |
| Aachen _West Germany_ | 190 | 16.9% [14.8%, 19.2%] |
| Osnabrueck _West Germany_ | 178 | 15.8% [13.8%, 18.1%] |
| Reutlingen _West Germany_ | 117 | 10.4% [8.8%, 12.3%] |
| Freiburg _West Germany_ | 139 | 12.4% [10.6%, 14.4%] |
| Greifswald _East Germany_ | 164 | 14.6% [12.6%, 16.8%] |
| Chemnitz _East Germany_ | 128 | 11.4% [9.7%, 13.4%] |
| Magdeburg _East Germany_ | 208 | 18.5% [16.3%, 20.9%] |
| **Gender** | | |
| Female | 696 | 61.9% [59%, 64.7%] |
| Male | 428 | 38.1% [35.3%, 41%] |
| **Age groups** | | |
| < 40 | 161 | 14.3% [12.4%, 16.5%] |
| 40-59 | 493 | 43.9% [41%, 46.8%] |
| ≥ 60 | 470 | 41.8% [39%, 44.7%] |
| ^1^Total amount of study participants who filled out the questionnaire about poliomyelitis vaccination and were able to match to their MuSPAD; ^²^showing the frequency and 95% CI; * classified according to ISCED (International Standard Classification of Education); level 3 equals German “Abitur” | | |

**Supplement Table 2** Characteristics of study population (unweighted frequencies)

| **Characteristics** | **Overall** | | **Recalled and documented vaccination uptake^3^** | | **Documented**  **Vaccination uptake** | | **Recalled vaccination uptake** | |
| --- | --- | --- | --- | --- | --- | --- | --- | --- |
|  | **Total N*^1^*** | **% [95% CI]^2^** | **Total N*^1^*** | **% [95% CI]^2^** | **Total N*^1^*** | **% [95% CI]^2^** | **Total N^1^** | **% [95% CI]^2^** |
| **Total** | 1,124 | 100% | 1,056 | 94% [92.4%, 95.2%] | 823 | 73.2% [70.6%, 75.7%] | 233 | 20.7% [18.5%, 23.2%] |
| **Region of Germany^3^** | | | | |  |  |  |  |
| West Germany | 624 | 55.5% [52.6%, 58.4%] | 584 | 55.3% [52.3%, 58.3%] | 455 | 55.3% [51.9%, 58.7%] | 129 | 55.4% [48.9%, 61.6%] |
| East Germany | 500 | 44.5% [41.6%, 47.4%] | 472 | 44.7% [41.7%, 47.7%] | 368 | 44.7% [41.3%, 48.1%] | 104 | 44.6% [38.4%, 51.1%] |
| **Age groups** | | | | |  |  |  |  |
| <40 | 161 | 14.3% [12.4%, 16.5%] | 148 | 14% [12.1%, 16.2%] | 138 | 16.8% [14.4%, 19.5%] | 10 | 4.3% [2.3%, 7.7%] |
| 40-59 | 493 | 43.9% [41%, 46.8%] | 468 | 44.3% [41.3%, 47.3%] | 375 | 45.6% [42.2%, 49%] | 93 | 39.9% [33.8%, 46.3%] |
| ≥60 | 470 | 41.8% [39%, 44.7%] | 440 | 41.7% [38.7%, 44.7%] | 310 | 37.7% [34.4%, 41%] | 130 | 55.8% [49.4%, 62%] |
| **Gender** | | | | |  |  |  |  |
| Male | 428 | 38.1% [35.3%, 41%] | 398 | 37.7% [34.8%, 40.7%] | 294 | 35.7% [32.5%, 39.1%] | 104 | 44.6% [38.4%, 51.1%] |
| Female | 696 | 61.9% [59%, 64.7%] | 658 | 62.3% [59.3%, 65.2%] | 529 | 64.3% [60.9%, 67.5%] | 129 | 55.4% [48.9%, 61.6%] |
| **Education** | | | | |  |  |  |  |
| Lower than ISCED level 3* | 341 | 30.3% [27.7%, 33.1%] | 309 | 29.3% [26.6%, 32.1%] | 236 | 28.7% [25.7%, 31.9%] | 73 | 31.3% [25.7%, 37.5%] |
| ISCED level 3* | 770 | 68.5% [65.7%, 71.2%] | 734 | 69.5% [66.7%, 72.2%] | 577 | 70.1% [66.9%, 73.1%] | 157 | 67.4% [61.1%, 73.1%] |
| Missing/Other | 13 | 1.2% [0.7%, 2%] | 13 | 1.2% [0.7%, 2.1%] | 10 | 1.2% [0.7%, 2.2%] | 3 | 1.3% [0.4%, 3.7%] |
| **Vaccination records available** | | | | |  |  |  |  |
| No | 27 | 2.4% [1.7%, 3.5%] | 20 | 1.9% [1.2%, 2.9%] | - | - | 20 | 8.6% [5.6%, 12.9%] |
| Yes | 1,097 | 97.6% [96.5%, 98.3%] | 1,036 | 98.1% [97.1%, 98.8%] | 823 | 100% [99.5%, 100%] | 213 | 91.4% [87.1%, 94.4%] |
| ^1^Total amount of study participants who filled out the questionnaire about poliomyelitis vaccination and were able to match to their MuSPAD; ^²^showing the frequency and 95% CI; * classified according to ISCED (International Standard Classification of Education); level 3 equals German “Abitur”; ^3^The regions of Germany correspond to the former division into the German Democratic Republic (GDR) and the Federal Republic of Germany (FRG). | | | | | | | | |

**Supplement Table 3:** Vaccination uptake against polio in the PCR-4-ALL/ MuSPAD cohort

| Overall – Vaccination uptake | Counts (n)^1^ | Frequency % (95% CI)  -unweighted-^2^ | Frequency % (95% CI)  -weighted-^3^ |
| --- | --- | --- | --- |
| Documented vaccination uptake | 823 | 73.2% [70.6%, 75.7%] | 74.3% [71.1%, 77.5%] |
| Recalled vaccination uptake | 233 | 20.7% [18.5%, 23.2%] | 19.0% [16.2%, 21.7%] |
| No report on vaccination uptake | 68 | 6.2% [4.8%, 7.6%] | 6.7% [4.8%, 8.6%] |

| Age groups | Immune status | Counts (n) | Frequency % (95% CI)  -unweighted-^2^ | Frequency % (95% CI)  -weighted-^3^ |
| --- | --- | --- | --- | --- |
| < 40 | Documented vaccination uptake | 138 | 85.7% [79.5%, 90.3%] | 86.7% [80.6%, 92.8%] |
| < 40 | Recalled vaccination uptake | 10 | 6.2% [3.4%, 11.1%] | 5.7% [1.4%, 10%] |
| < 40 | No report on vaccination uptake | 13 | 8.1% [4.8%, 13.3%] | 7.6% [3%, 12.2%] |
| 40-59 | Documented vaccination uptake | 375 | 76.1% [72.1%, 79.6%] | 73.8% [69.1%, 78.5%] |
| 40-59 | Recalled vaccination uptake | 93 | 18.9% [15.7%, 22.6%] | 20.6% [16.2%, 24.9%] |
| 40-59 | No report on vaccination uptake | 25 | 5.1% [3.5%, 7.4%] | 5.6% [3.2%, 8.1%] |
| ≥ 60 | Documented vaccination uptake | 310 | 66% [61.6%, 70.1%] | 64.6% [59.4%, 69.9%] |
| ≥ 60 | Recalled vaccination uptake | 130 | 27.7% [23.8%, 31.9%] | 28.4% [23.4%, 33.4%] |
| ≥ 60 | No report on vaccination uptake | 30 | 6.4% [4.5%, 9%] | 7% [4.1%, 9.9%] |
| Gender | **Vaccination uptake** | **Counts (n)**^1^ | **Frequency % (95% CI)**  -unweighted-^2^ | **Frequency % (95% CI)**  -weighted-^3^ |
| Female | Documented vaccination uptake | 529 | 76% [72.7%, 79%] | 69.7% [64.5%, 74.9%] |
| Female | Recalled vaccination uptake | 129 | 18.5% [15.8%, 21.6%] | 23.4% [18.7%, 28.1%] |
| Female | No report on vaccination uptake | 38 | 5.6% [4%, 7.4%] | 6.9% [4%, 9.7%] |
| Male | Documented vaccination uptake | 294 | 68.7% [64.1%, 72.9%] | 78.8% [75.1%, 82.5%] |
| Male | Recalled vaccination uptake | 104 | 24.2% [20.5%, 28.6%] | 14.6% [11.6%, 17.6%] |
| Male | No report on vaccination uptake | 30 | 7% [5%, 9.8%] | 6.6% [4%, 9.2%] |
| ^1^Total amount of study participants who filled out the questionnaire about poliomyelitis vaccination and were able to match to their MuSPAD; ^2^showing the -unweighted- (by age, gender, region) frequency and 95% CI;^3^ showing the -weighted- (by age, gender, region) frequency and 95% CI | | | | |


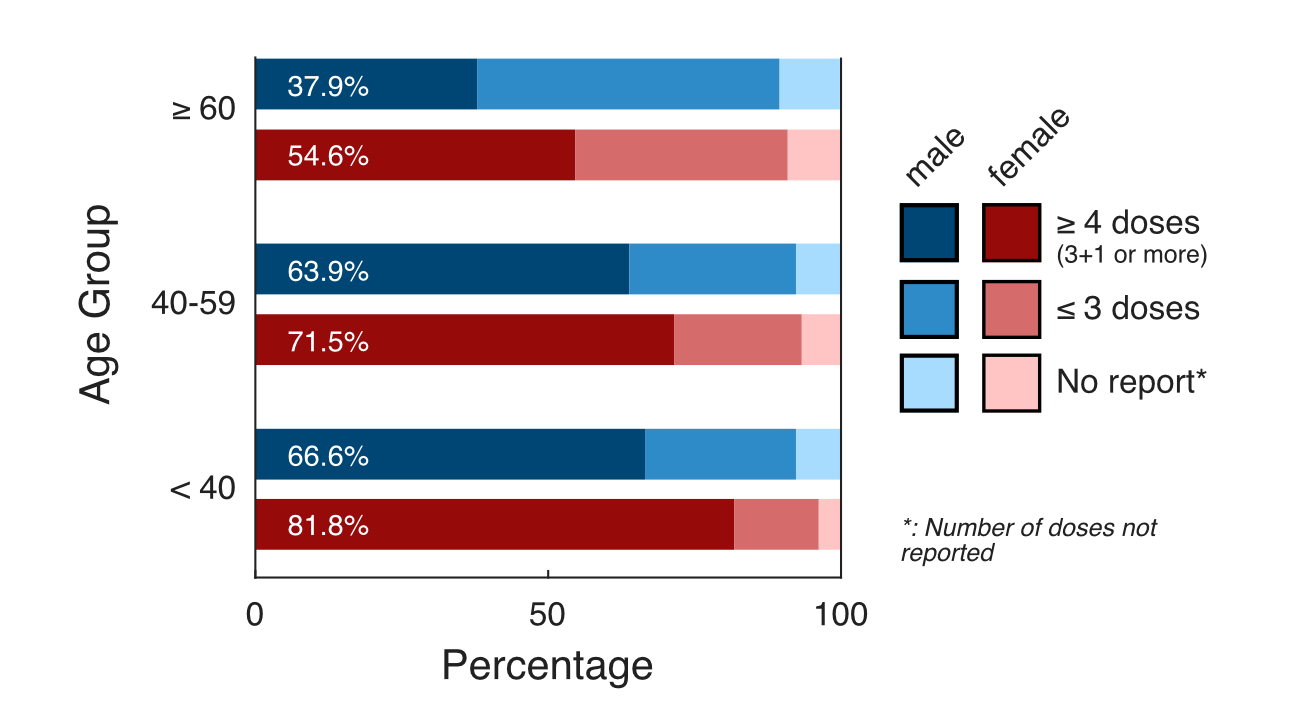


Supplement Figure 2 Weighted frequency: Documented poliomyelitis immunization status in the PCR-4-ALL/MuSPAD cohorts based on vaccination record according to the amount of doses (≤ 3 doses / ≥ 4 doses); (n=823)

**Supplement Table 4:** Stratification of poliomyelitis vaccination by age groups in those with a vaccination certificate based on the 2+1 vaccination schema

| Characteristics | Vaccine uptake | Counts (n)^1^ | Frequency % (95% CI)  -unweighted-^2^ | Frequency % (95% CI)  -weighted-^3^ |
| --- | --- | --- | --- | --- |
| Overall | No report number of doses | 67 | 8.1% [6.5%, 10.2%] | 7.3% [5.0%, 9.6%] |
|  | *≤ 2 doses* | 164 | 19.9% [17.3%, 22.8%] | 17.6% [14.2%, 20.9%] |
|  | ≥ 3 doses | 592 | 71.9% [68.8%, 74.9%] | 75.1% [71.3%, 79.0%] |
| Age groups | **Vaccine uptake** | **Counts (n)**^1^ | **Frequency % (95% CI)**  -unweighted-^2^ | **Frequency % (95% CI)**  -weighted-^3^ |
| < 40 | No report number of doses | 7 | 5.1% [2.5%, 10.1%] | 5.3% [0.6%, 10%] |
| < 40 | *≤ 2 doses* | 11 | 8% [4.5%, 13.7%] | 10.9% [4.2%, 17.6%] |
| < 40 | ≥ 3 doses | 120 | 87% [80.3%, 91.6%] | 83.8% [76.1%, 91.6%] |
| 40-59 | No report number of doses | 31 | 8.3% [5.9%, 11.5%] | 7.1% [4.1%, 10.0%] |
| 40-59 | *≤ 2 doses* | 52 | 13.9% [10.7%, 17.7%] | 13.1% [9.0%, 17.3%] |
| 40-59 | ≥ 3 doses | 292 | 77.9% [73.4%, 81.8%] | 79.8% [75.0%, 84.6%] |
| ≥ 60 | No report number of doses | 29 | 9.4% [6.6%, 13.1%] | 9.8% [5.8%, 13.8%] |
| ≥ 60 | *≤ 2 doses* | 101 | 32.6% [27.6%, 38%] | 29.5% [23.4%, 35.5%] |
| ≥ 60 | ≥ 3 doses | 180 | 58.1% [52.5%, 63.4%] | 60.7% [54.2%, 67.2%] |
| Gender | **Vaccine uptake** | **Counts (n)**^1^ | **Frequency % (95% CI)**  -unweighted-^2^ | **Frequency % (95% CI)**  -weighted-^3^ |
| Male | No report number of doses | 30 | 10.2% [7.2%, 14.2%] | 8.7% [4.6%, 12.8%] |
| Male | *≤ 2 doses* | 80 | 27.2% [22.4%, 32.6%] | 24.9% [18.7%, 31.2%] |
| Male | ≥ 3 doses | 184 | 62.6% [56.9%, 67.9%] | 66.4% [59.5%, 73.2%] |
| Female | No report number of doses | 37 | 7% [5.1%, 9.5%] | 6.1% [3.7%, 8.6%] |
| Female | *≤ 2 doses* | 84 | 15.9% [13%, 19.2%] | 11.2% [8.1 %, 14.3%] |
| Female | ≥ 3 doses | 408 | 77.1% [73.4%, 80.5%] | 82.7% [78.9%, 86.4%] |
| ^1^Total amount of study participants who filled out the questionnaire about poliomyelitis vaccination and were able to match to their MuSPAD; ^2^showing the -unweighted- (by age, gender, region) frequency and 95% CI; ^3^showing the -weighted- (by age, gender, region) frequency and 95% CI | | | | |

**Supplement Table 5:** Stratification of poliomyelitis vaccination by age groups in those with a vaccination certificate based on the 3+1 vaccination schema

| Characteristics | Vaccine uptake | Counts (n)^1^ | Frequency % (95% CI)  -unweighted-^2^ | Frequency % (95% CI)  -weighted-^3^ |
| --- | --- | --- | --- | --- |
| Overall | No report number of doses | 67 | 8.1% [6.5%, 10.2%] | 7.3% [5.0%, 9.6%] |
|  | *≤ 3 doses* | 272 | 33% [29.9%, 36.3%] | 29.2% [25.2%, 33.2%] |
|  | ≥ 4 doses | 484 | 58.8% [55.4%, 62.1%] | 63.5 % [59.2%, 67.8%] |
| Age groups | **Vaccine uptake** | **Counts (n)**^1^ | **Frequency % (95% CI)**  -unweighted-^2^ | **Frequency % (95% CI)**  -weighted-^3^ |
| < 40 | No report number of doses | 7 | 5.1% [2.5%, 10.1%] | 5.3% [0.6%, 10.0%] |
| < 40 | *≤ 3 doses* | 23 | 16.7% [11.4%, 23.8%] | 18.7% [10.8%, 26.6%] |
| < 40 | ≥ 4 doses | 108 | 78.3% [70.7%, 84.3%] | 76.0% [67.3%, 84.6%] |
| 40-59 | No report number of doses | 31 | 8.3% [5.9%, 11.5%] | 7.1% [4.1%, 10.0%] |
| 40-59 | *≤ 3 doses* | 96 | 25.6% [21.4%, 30.2%] | 24.7% [19.5%, 29.9%] |
| 40-59 | ≥ 4 doses | 248 | 66.1% [61.2%, 70.7%] | 68.2% [62.6%, 73.8%] |
| ≥ 60 | No report number of doses | 29 | 9.4% [6.6%, 13.1%] | 9.8% [5.8%, 13.8%] |
| ≥ 60 | *≤ 3 doses* | 153 | 49.4% [43.8%, 54.9%] | 45.2% [38.5%, 51.9%] |
| ≥ 60 | ≥ 4 doses | 128 | 41.3% [35.9%, 46.8%] | 45.0% [38.3%, 51.6%] |
| Gender | **Vaccine uptake** | **Counts (n)**^1^ | **Frequency % (95% CI)**  **-**unweighted-^2^ | **Frequency % (95% CI)**  -weighted-^3^ |
| Male | No report number of doses | 30 | 10.2% [7.2%, 14.2%] | 8.7% [4.6%, 12.8%] |
| Male | *≤ 3 doses* | 122 | 41.5% [36%, 47.2%] | 37.0% [30.1%, 43.9%] |
| Male | ≥ 4 doses | 142 | 48.3% [42.6%, 54%] | 54.3% [47.1%, 61.5%] |
| Female | No report number of doses | 37 | 7% [5.1%, 9.5%] | 6.1% [3.7%, 8.6%] |
| Female | *≤ 3 doses* | 150 | 28.4% [24.7%, 32.3%] | 22.4% [18.1%, 26.8%] |
| Female | ≥ 4 doses | 342 | 64.7% [60.5%, 68.6%] | 71.4% [66.7%, 76.2%] |
| ^1^Total amount of study participants who filled out the questionnaire about poliomyelitis vaccination and were able to match to their MuSPAD; ^2^showing the -unweighted- (by age, gender, region) frequency and 95% CI; ^3^showing the -weighted- (by age, gender, region) frequency and 95% CI | | | | |

**Supplement Table 6** Frequency of poliomyelitis vaccine in vaccination records

| Number of polio vaccine doses | Counts (n)^1^ | Frequency % (95% CI)  -unweighted-^2^ | Frequency % (95% CI)  -weighted-^3^ |
| --- | --- | --- | --- |
| 1 | 57 | 7.5% [5.9%, 9.6%] | 7.3% [4.8%, 9.7%] |
| 2 | 107 | 14.2% [11.8%, 16.8%] | 11.7% [8.7%, 14.6%] |
| 3 | 108 | 14.3% [12%, 17%] | 12.5% [9.6%, 15.4%] |
| 4 | 108 | 14.3% [12%, 17%] | 17.4% [13.6%, 21.2%] |
| 4+ | 376 | 49.7% [46.2%, 53.3%] | 51.1% [46.5%, 55.8%] |
| Missing | 67 | NA | NA |
| ^1^Total amount of study participants who filled out the questionnaire about poliomyelitis vaccination and were able to match to their MuSPAD; ^2^showing the -unweighted- (by age, gender, region) frequency and 95% CI; ^3^showing the -weighted- (by age, gender, region) frequency and 95% CI | | | |

**Supplement Table 7:** Overview of participants’ receiving the first poliomyelitis vaccine doses before the second year of life

| **Groups** | **Counts (n)**^1^ | **Frequency % (95% CI)**  -unweighted-^2^ | **Frequency % (95% CI)**  -weighted-^3^ |
| --- | --- | --- | --- |
| **Total** | 397/686 | 57.9% [54.1%, 61.5%] | 62.5% [57.9%, 67.0%] |
| **Age groups** | | |  |
| <40 | 112/124 | 90.3% [83.8%, 94.4%] | 88.8% [82.1%, 95.6%] |
| 40-59 | 236/318 | 74.2% [69.1%, 78.7%] | 75.5% [70.0%,81.0%] |
| ≥60 | 49/244 | 20.1% [15.5%, 25.6%] | 14.8% [9.8%, 19.9%] |
| **Gender** | | |  |
| Female | 289/456 | 63.4% [58.9%, 67.7%] | 69.8% [64.7%, 74.8%] |
| Male | 108/230 | 47.0% [40.6%, 53.4%] | 53.1% [45.1%, 61.1%] |
| ^1^Total amount of study participants who filled out the questionnaire about poliomyelitis vaccination and were able to match to their MuSPAD; ^2^showing the -unweighted- (by age, gender, region) frequency and 95% CI; ^3^showing the -weighted- (by age, gender, region) frequency and 95% CI | | | |

**Supplement Table 8** Numbers of poliomyelitis vaccine types stratified by groups and doses

| Dose number | Dose type | Counts (n)^1^ | Frequency % (95% CI)  -unweighted-^2^ | Frequency % (95% CI)  -weighted-^3^ |
| --- | --- | --- | --- | --- |
| Overall | IPV | 704 | 25.4% [23.8%, 27.1%] | 26.2% [24.0%, 28.3%] |
| Overall | OPV | 898 | 32.4% [30.7%, 34.2%] | 29.9% [27.8%, 32.0%] |
| Overall | Unidentifiable in the records (by participants) | 1166 | 42.1% [40.3%, 44%] | 43.9% [41.5%, 46.4%] |
| Dose 1 | IPV | 104 | 14.5% [12.1%, 17.2%] | 16.1% [12.5%, 19.7%] |
| Dose 1 | OPV | 287 | 39.9% [36.4%, 43.5%] | 36.7% [32.3%, 41.2%] |
| Dose 1 | Unidentifiable in the records (by participants) | 328 | 45.6% [42%, 49.3%] | 47.2% [42.4%, 52.0%] |
| Dose 2 | IPV | 125 | 18.7% [16%, 21.9%] | 16.2% [12.7%, 19.8%] |
| Dose 2 | OPV | 235 | 35.2% [31.7%, 38.9%] | 34.2% [29.6%, 38.7%] |
| Dose 2 | Unidentifiable in the records (by participants) | 307 | 46% [42.3%, 49.8%] | 49.6% [44.6%, 54.6%] |
| Dose 3 | IPV | 107 | 18.9% [15.9%, 22.4%] | 17.0% [13.1%, 20.8%] |
| Dose 3 | OPV | 204 | 36.1% [32.3%, 40.1%] | 33.8% [28.9%, 38.8%] |
| Dose 3 | Unidentifiable in the records (by participants) | 254 | 45% [40.9%, 49.1%] | 49.2% [43.8%, 54.5%] |
| Dose 4 | IPV | 129 | 28.1% [24.2%, 32.4%] | 32.1% [26.5%, 37.8%] |
| Dose 4 | OPV | 131 | 28.5% [24.6%, 32.8%] | 24.8% [20.2%, 29.4%] |
| Dose 4 | Unidentifiable in the records (by participants) | 199 | 43.4% [38.9%, 47.9%] | 43.1% [37.2%, 49.0%] |
| Dose > 4 | IPV | 239 | 66.8% [61.7%, 71.4%] | 70.1% [64.4%, 75.8%] |
| Dose > 4 | OPV | 41 | 11.5% [8.6%, 15.2%] | 9.6% [6.1%, 13.1%] |
| Dose > 4 | Unidentifiable in the records (by participants) | 78 | 21.8% [17.8%, 26.4%] | 20.3% [15.3%, 25.2%] |
| ^1^Total amount of study participants who filled out the questionnaire about poliomyelitis vaccination and were able to match to their MuSPAD; ^2^showing the -unweighted- (by age, gender, region) frequency and 95% CI; ^3^showing the -weighted- (by age, gender, region) frequency and 95% CI | | | | |

**Supplement Table 9** Odds Ratios of the determinants of recalled vs documented vaccination uptake (n=1,043), considering survey weights

| Characteristic (n)^1^ | Odds Ratio | 95% CI | p-value |
| --- | --- | --- | --- |
| Age groups | | | |
| <40 (n=146) | Reference | | |
| 40-59 (n=464) | 3.93 | 1.67, 9.24 | 0.002 |
| ≥ 60 (n=433) | 5.89 | 2.48, 14.01 | <0.001 |
| Gender | | | |
| Female (n=652) | Reference | | |
| Male (n=391) | 1.51 | 1.04, 2.20 | 0.03 |
| Education* | | | |
| ISCED level 3 (n=734) | Reference | | |
| Lower than ISCED level 3 (n=309) | 1,30 | 0.82, 2.05 | 0.26 |
| Region | |  |  |
| Eastern Germany (n=467) | Reference | | |
| Western Germany (n=576) | 1.12 | 0.81, 1.55 | 0.50 |
| *classified according to ISCED (International Standard Classification of Education); level 3 equals German “Abitur”; ^1^Total amount of study participants who filled out the questionnaire about poliomyelitis vaccination and were able to match to their MuSPAD with no missing observation | | | |
